# Enoyl-Coenzyme A Respiration via Formate Cycling in Syntrophic Bacteria

**DOI:** 10.1128/mbio.03740-21

**Published:** 2022-02-01

**Authors:** Michael Agne, Lena Appel, Carola Seelmann, Matthias Boll

**Affiliations:** a Faculty of Biology–Microbiology, Albert-Ludwigs-Universität Freiburg, Freiburg im Breisgau, Germany; b Spemann Graduate School of Biology and Medicine (SGBM), University of Freiburg, Freiburg, Germany; University of California, Irvine

**Keywords:** Syntrophus, syntrophy, redox loop, formate cycling, formate dehydrogenase, respiration

## Abstract

Syntrophic bacteria play a key role in the anaerobic conversion of biological matter to methane. They convert short-chain fatty acids or alcohols to H_2_, formate, and acetate that serve as substrates for methanogenic archaea. Many syntrophic bacteria can also grow with unsaturated fatty acids such as crotonate without a syntrophic partner, and the reducing equivalents derived from the oxidation of one crotonate to two acetate are regenerated by the reduction of a second crotonate. However, it has remained unresolved how the oxidative and reductive catabolic branches are interconnected and how energy may be conserved in the reductive branch. Here, we provide evidence that during axenic growth of the syntrophic model organism Syntrophus aciditrophicus with crotonate, the NAD^+^-dependent oxidation of 3-hydroxybutyryl-CoA to acetoacetyl-CoA is coupled to the reduction of crotonyl-CoA via formate cycling. In this process, the intracellular formate generated by a NAD^+^-regenerating CO_2_ reductase is taken up by a periplasmic, membrane-bound formate dehydrogenase that in concert with a membrane-bound electron-transferring flavoprotein (ETF):methylmenaquinone oxidoreductase, ETF, and an acyl-CoA dehydrogenase reduces intracellular enoyl-CoA to acyl-CoA. This novel type of energy metabolism, referred to as enoyl-CoA respiration, generates a proton motive force via a methylmenaquinone-dependent redox-loop. As a result, the beneficial syntrophic cooperation of fermenting bacteria and methanogenic archaea during growth with saturated fatty acids appears to turn into a competition for formate and/or H_2_ during growth with unsaturated fatty acids.

## INTRODUCTION

The degradation of biomass into methane by anaerobic microorganisms is an essential process in the global carbon cycle but also for the formation of renewable biogas at engineered systems (1, 2). It involves (i) the hydrolysis of biopolymers and their conversion into primary fermentation products such as short-chain fatty acids (scFA) and alcohols; (ii) the oxidation of the latter by secondary fermenting bacteria to acetate coupled to the reduction of CO_2_/protons to formate/H_2_, respectively; and (iii) the formation of methane by methanogenic archaea from H_2_ + CO_2_, formate or acetate. Alternatively, acetate may be formed by acetogenic bacteria from various organic substrates or from H_2_ + CO_2_ (3). In secondary fermenting bacteria, the oxidation of the saturated scFA model compound butyrate to two acetate is coupled to proton or CO_2_ reduction. This process is endergonic under standard conditions (butyrate^–^ + 2 H_2_O → 2 acetate^–^ + H^+^ + H_2_; ΔG°’ = +48 kJ mol^−1^), but becomes exergonic at low H_2_ partial pressures (≤10^−4 ^atm) or low formate concentrations (≤10 μM). The low partial pressure/concentration values are maintained by methanogenic archaea that efficiently capture formate and/or H_2_ during methane production resulting in E’-values ≈ −290 mV for the 2H^+^/H_2_ and CO_2_ + H^+^/formate couples. Thus, the syntrophic interspecies electron transfer between secondary fermenting bacteria and methanogenic archaea is essential for methane formation from primary fermentation products (2, 4–7).

The syntrophic oxidation of butyrate to two acetate proceeds via standard β-oxidation (8). Briefly, the activated butyryl-coenzyme A (CoA) intermediate is oxidized first to crotonyl-CoA by an acyl-CoA dehydrogenase (DH) with an ETF serving as acceptor (E°’ ≈ −10 mV) (9, 10) and then, after hydration to 3-hydroxybutyryl-CoA, to acetoacetyl-CoA by an NAD^+^-dependent 3-hydroxyacyl-CoA DH (E°’ ≈ −250 mV) (6). Thiolytic cleavage gives two acetyl-CoA from which one is used for butyrate activation, whereas the second generates one ATP via substrate level phosphorylation (SLP). The electrons derived from 3-hydroxyacyl-CoA DH reaction are proposed to be transferred at a redox potential that is sufficiently negative for H^+^/CO_2_ reduction via transiently formed NADH. In agreement, there is evidence that non-electron-bifurcating hydrogenases are involved in NADH-dependent proton reduction in syntrophic bacteria (11, 12). In contrast, the electron transfer from acyl-CoA to H^+^/CO_2_ is highly endergonic (ΔG ≈ +54 kJ mol^−1^). Multiple omics-based studies predicted that a reverse redox-loop is involved in the electron transfer from acyl-CoA DH via ETF to membrane-bound hydrogenases or formate DHs (FDHs) (13–16). Recently, biochemical evidence for such a redox-loop was presented in studies with the deltaproteobacterium Syntrophus aciditrophicus (17). It involves a membrane-bound diheme/FeS cluster containing electron-transferring flavoprotein (ETF):methylmenaquinone oxidoreductase (EMO) that transfers electrons from reduced ETF (ETF_red_) to 8-methylmenaquinone (8-MMK). The reduced 8-MMKH_2_ is then reoxidized by a membrane-bound FDH (mFDH). The opposite orientation of mFDH (periplasmic) and EMO (cytoplasmic) allows for the proton motive force (pmf) driven reverse electron transfer from acyl-CoA to CO_2_. The reduction potentials of the high- and low-potential two heme *b* cofactors of EMO (–80 and −220 mV, respectively) and MMK (–150 kJ mol^−1^) perfectly fit to a membrane potential driven reverse redox loop at the expense of two protons transported to the periplasm per electron transferred (17).

**FIG 1:**
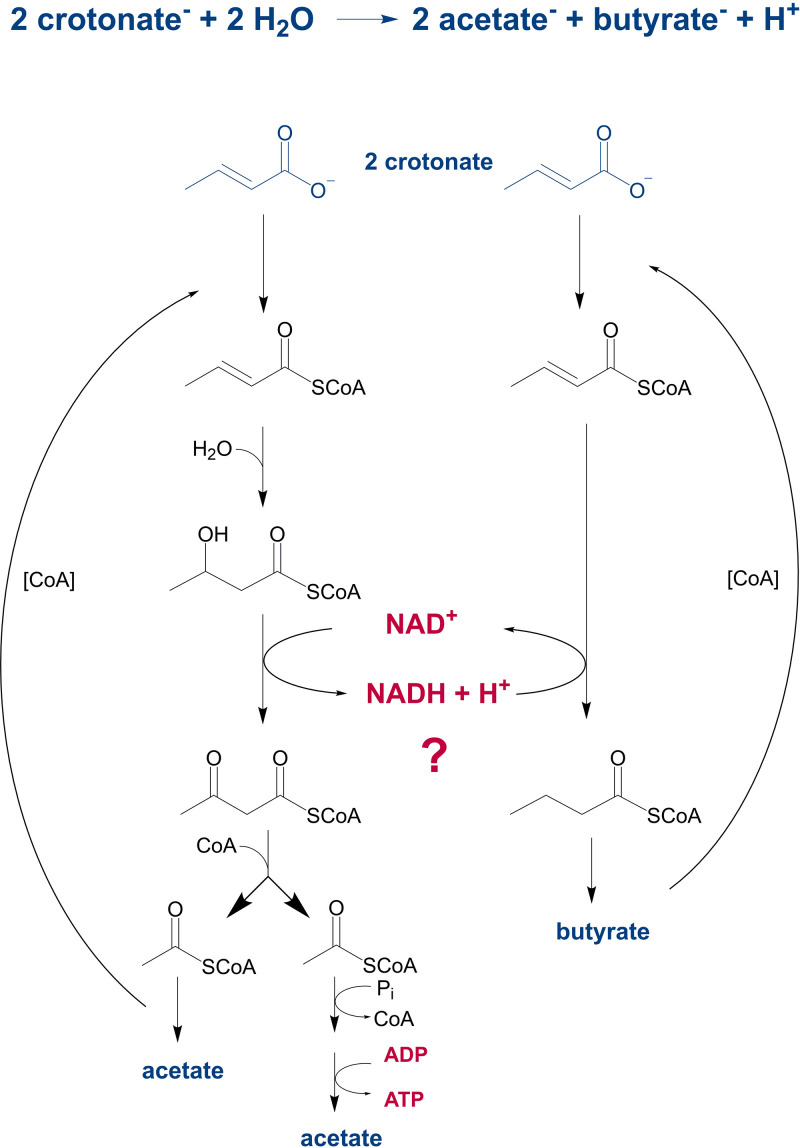
General scheme for crotonate degradation in fermenting bacteria in the absence of a syntrophic partner. The NAD^+^ consumed in the oxidative branch (left panel) is regenerated by the reduction of a second crotonyl-CoA to butyryl-CoA. For easier presentation, only CoA transferases are shown as crotonyl-CoA forming enzymes. Alternatively, ATP-dependent acyl-CoA synthethases may be involved. ATP synthesis via SLP, here shown via transacetylase and acetate kinase, may alternatively involve a pyrophosphate-dependent CoA ligase (28).

Many secondary fermenting bacteria can grow with unsaturated scFAs such as crotonate without a syntrophic partner with one crotonate being oxidized to two acetate and a second one being reduced to butyrate (Fig. 1) (13, 18, 19). In the oxidative branch, the NAD^+^-dependent 3-hydroxybutyryl-CoA DH catalyzes the only oxidation step. The NAD^+^ formed is regenerated by the NADH-dependent reduction of a second crotonyl-CoA to butyryl-CoA. Though this pathway looks, at first view, like a standard fermentation process that conserves energy exclusively via SLP (0.5 ATP/crotonate) (20), it has remained unknown how the oxidative and reductive branches are linked during axenic growth of syntrophs with crotonate. The reduction of crotonyl-CoA to butyryl-CoA (E°’ = −10 mV) by NADH (E°’ = −320 mV) is highly exergonic under standard conditions (≈ −60 kJ mol^−1^) and is in the range of the cellular Gibbs free energy of ATP hydrolysis (21). Thus, the question arises whether and how this reaction could allow for additional energy conservation in syntrophic bacteria, which would substantially increase the ATP yield. There are three possibilities for an energetic coupling during NADH-dependent enoyl-CoA reduction. (i) An electron-bifurcating ETF may couple the exergonic reduction of enoyl-CoA by NADH to the endergonic reduction of ferredoxin (Fd) by NADH. Such a process has been described in many fermenting Firmicutes (22–24). A membrane-bound Rnf complex (an energy-conserving Fd_red_:NAD^+^ oxidoreductase) may then couple the exergonic reoxidization of Fd_red_ by NAD^+^ to the transport of protons or sodium ions from the cyto- to the periplasm (25). (ii) A respiratory, proton-pumping NADH:8-MMK oxidoreductase may transfer electrons from NADH to the 8-MMK pool, and the 8-MMKH_2_ formed could be reoxidized by enoyl-CoA involving EMO, a non-bifurcating ETF and an acyl-CoA DH. (iii) A pmf could be generated via a redox-loop involving cytoplasmic, NADH-dependent formate/H_2_ forming and periplasmic, membrane-bound 8-MMK-dependent formate/H_2_ oxidizing oxidoreductases. Reduced 8-MMKH_2_ could serve as donor for crotonyl-CoA reduction involving EMO, ETF, and acyl-CoA DH. In a variant of such a process, cytoplasmic CO_2_ reduction may be accomplished by a Fd_red_-dependent FDH, providing that Fd is reduced by the NADH formed in the oxidative branch via a Rnf complex.

Here, we investigated the unknown energetic coupling of the oxidative and reductive branches of axenic crotonate catabolism in the syntrophic model organism Syntrophus aciditrophicus. We provide evidence that energy conservation proceeds to a major extent via respiratory electron transport phosphorylation (ETP) with acyl-CoA DHs serving as terminal reductases. We refer to this previously unknown respiration-type as enoyl-CoA respiration that proceeds via formate cycling.

## RESULTS

### Axenic crotonate degradation in Syntrophus aciditrophicus as model system.

**FIG 2:**
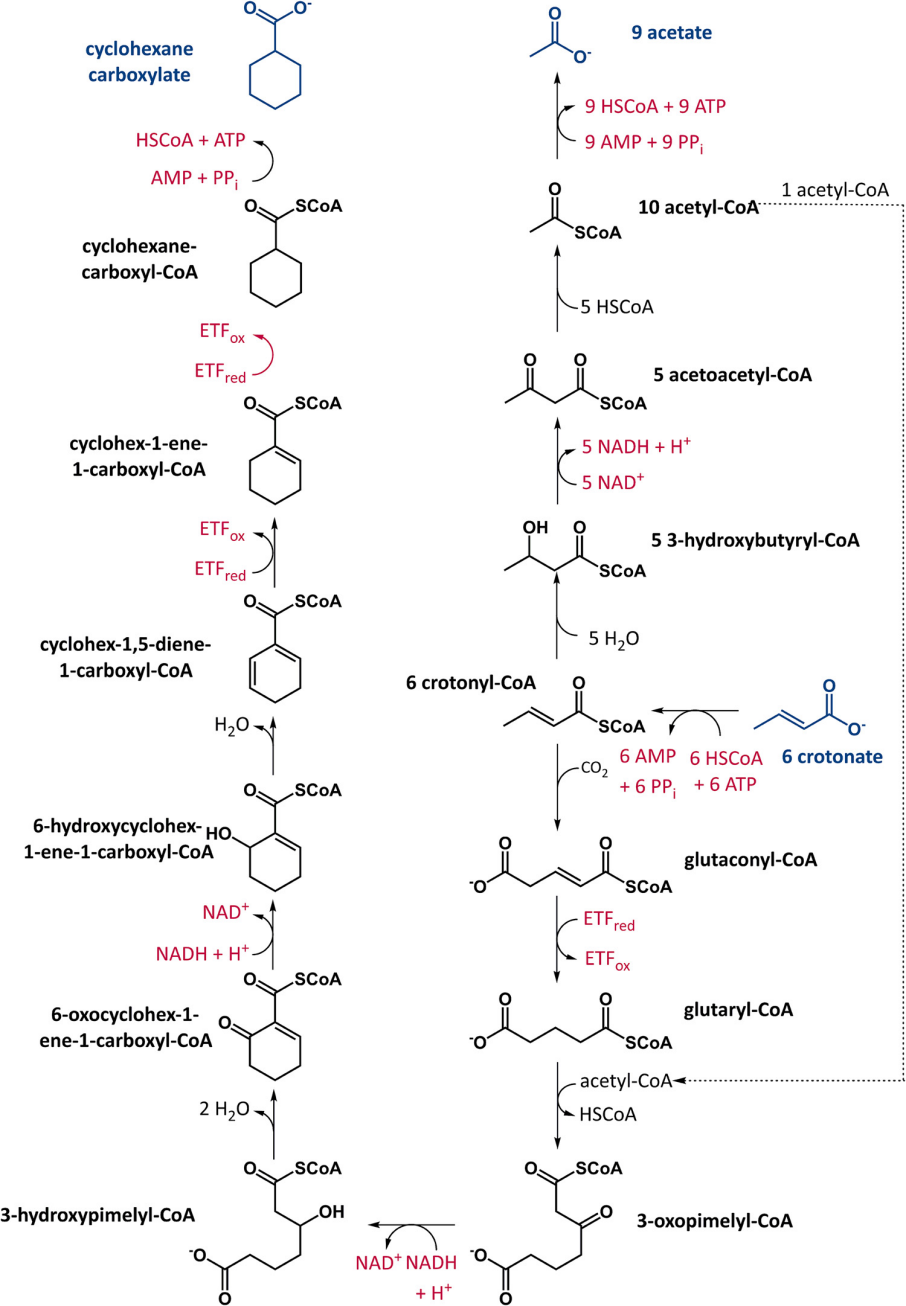
Axenic crotonate degradation pathway of S. aciditrophicus. Five molecules crotonyl-CoA are oxidized to 10 molecules acetyl-CoA, one of the latter is used for reverse thiolase reaction in the reductive branch. The five NADH formed are directly reoxidized by two NADH-dependent 3-hydroxyacyl-CoA dehydrogenases and indirectly by three ETF-dependent acyl-CoA dehydrogenases (27).

To investigate the link between the oxidative and reductive branches during axenic growth with an unsaturated scFA, we opted for crotonate degradation in Syntrophus aciditrophicus as the model system. The fermentation balances and the intermediates of this pathway have been elucidated previously in this organism (26, 27). Further, it can be grown easily with crotonate in the 200-L-scale in the absence of a methanogen yielding ≈200 g of wet cell mass within 2 weeks (17). The reductive branch of axenic crotonate degradation in S. aciditrophicus differs from the canonical pathway depicted in Fig. 1 as it does not proceed via the one-step regeneration of NAD^+^ by butyryl-CoA DH. Instead, it involves chain elongation and a series of reversed β-oxidation-like steps including both, reduced ETF- and NADH-dependent DHs finally yielding cyclohexanecarboxylate as excreted end product (Fig. 2). In axenically grown S. aciditrophicus, crotonate is degraded according to the equation (26):

$${\text{6 crotonate}}^{–}+{\text{ HCO}}_{\text{3}}{}^{–}+{\text{ 5 H}}_{\text{2}}\text{O}\to {\text{9 acetate}}^{–}+{\text{ cyclohexanecarboxylate}}^{–}+{\text{ 3 H}}^{+}$$

$$\Delta \text{G}{}^{\circ}’\;=\;–\text{48}\;{\text{kJ mol}}^{-\text{1}}$$

Five crotonate are oxidized to 10 acetyl-CoA producing five NADH in the oxidative branch, while reduction of a sixth crotonate regenerates the reducing equivalents (Fig. 2). Two NAD^+^ are directly regenerated by two NADH-dependent 3-hydroxyacyl-CoA DH, whereas the remaining three NADH are reoxidized by ETF-dependent acyl-CoA DH. One of the 10 acetyl-CoA formed in the oxidative branch is used in the reductive branch for chain length extension via a reversely operating β-ketothiolase.

This established pathway suggests that for the six crotonate converted, four net ATP are synthesized via SLP by pyrophosphate (PP_i_)- and AMP-dependent acetyl-CoA synthetases (28). This ATP yield is reduced by the ion motif force driven carboxylation of crotonyl-CoA to glutaconyl-CoA (1 Na^+^ translocated to the cytoplasm per six crotonate converted) in the reductive branch (29). Assuming that ATP synthase translocates three to four H^+^ per ATP synthesized, the overall yield is 3.7 ATP per cyclohexanecarboxylate formed giving around 0.6 ATP per crotonate consumed. However, the calculated free energy value of crotonate fermentation (–48 kJ mol^−1^ per crotonate) would allow for a higher ATP yield, and the experimentally determined growth yield (>1 ATP per crotonate [26]) is substantially higher. These findings raise the question whether and how energy may be conserved by the transfer of electrons from NADH to the three acyl-CoA DHs acting on glutaconyl-CoA (29), cyclohexa-1,5-diene-1-carboxyl-CoA, and cyclohex-1-ene-1-carboxyl-CoA (30) (Fig. 2).

### Genomic clues for possible NAD^+^ regeneration scenarios during axenic growth of S. aciditrophicus with crotonate.

We analyzed the genome of S. aciditrophicus for candidate genes that may be involved in regenerating the NAD^+^ reduced in the oxidative branch. The genome of S. aciditrophicus contains a single copy of *etfAB* genes (SYN_RS12520/RS12525). The corresponding ETF has recently been purified and unambiguously characterized as a non-electron-bifurcating ETF with an only marginal NADH:acceptor oxidoreductase activity, which rules out a role in NAD^+^ regeneration (17). Further, the genome does not contain genes encoding for the membrane-bound subunits of NADH:quinone oxidoreductase (respiratory complex I) (15).

The presence of two soluble NADH-dependent FDHs ([sFDHs], FDH-2 and FDH-4) and a NADH-dependent [Fe-Fe]-hydrogenase (HydAB) has been reported in genomic and proteomic studies (15, 31). The latter has been characterized after heterologous expression of the encoding genes as a non-electron-bifurcating, NADH-dependent enzyme (12). In HydB, conserved amino acid sequence motifs were identified near the NADH and FMN binding sites and the soluble-ligand-binding-beta-grasp (SLBB) domain that distinguish non-electron-bifurcating HydBs, and related NADH-binding subunits from FDHs from those of electron-bifurcating enzymes (12). We analyzed these distinguishing amino acid signatures in FdhB-2/FdhB-4 from S. aciditrophicus and identified them as characteristic for non-electron-bifurcating enzymes (Fig. S1). Further, FdhB-2 and FdhB-4 miss the C-terminal domain binding two [4Fe-4S] clusters typically found in electron-bifurcating FDHs/hydrogenases (12).

10.1128/mbio.03740-21.2 FIG S1 Distinguishing amino acid residues in the NADH/FMN-binding subunits of electron-bifurcating and non-electron-bifurcating FDHs/hydrogenases. The soluble NADH-dependent FDHs of S. aciditrophicus are highlighted by a frame. Other sequences were taken from Losey et al. (12). Download FIG S1, DOCX file, 0.1 MB. Copyright © 2022 Agne et al. 2022 Agne et al. https://creativecommons.org/licenses/by/4.0/ This content is distributed under the terms of the Creative Commons Attribution 4.0 International license.

The NADH/FMN binding subunits of hydrogenases are similar to the NADH/FMN-binding NuoF subunit of respiratory complex I (Escherichia coli notification). Using the experimentally verified HydB subunit from S. aciditrophicus, we used BLAST to screen the genome for potential NuoF-like NAD^+^-regenerating oxidoreductases (Table S1). Next to FdhB-2, FdhB-4, and HydB, three further candidates were identified with low expect values that were assigned to the NADH/FMN-binding BamH subunits of the class II benzoyl-CoA reductase (BCR) complex (32). This assignment is based on high similarities to experimentally characterized BamH subunits (32, 33), and by the presence of genes encoding other subunits of class II BCRs in direct vicinity of the bamH genes. Though class II BCRs exhibit NADH:viologen oxidoreductase activities that have been assigned to the BamH subunit (32), it is very unlikely that they play a significant role during crotonate fermentation because benzoyl-CoA is not a relevant intermediate of crotonate fermentation. The rather low expression of only one out of the three putative bamH genes during growth with crotonate is in line with this assumption (31). Finally, a gene product with similarities to the NADH/FMN-binding RnfC subunit of a putative membrane-bound Rnf complex was identified as potential NADH oxidizing enzyme, albeit with low similarities to NADH/FMN binding B-subunits of soluble hydrogenase and FDHs. Notably, the gene product was abundant in cells grown with crotonate (31), and an RnfC component from acetogenic *Acetobacterim woodii* was shown to exhibit a NADH:acceptor oxidoreductase activity (34). In summary, the genomic inventory of S. aciditrophicus suggests that NAD^+^-regeneration during crotonate fermentation may proceeds via non-electron bifurcating FDHs/hydrogenases, and/or a membrane-bound Rnf-complex. The involvement of electron-bifurcating ETFs, FDHs, hydrogenases, or a proton-pumping NADH:8-MMKH oxidoreductase can be rather ruled out.

10.1128/mbio.03740-21.1 TABLE S1 NuoF-like gene products in the genome of S. aciditrophicus. The HydB subunit of [Fe-Fe]-hydrogenase (SYN_RS05185) served as query sequence during BLAST application. Download Table S1, DOCX file, 0.02 MB. Copyright © 2022 Agne et al. 2022 Agne et al. https://creativecommons.org/licenses/by/4.0/ This content is distributed under the terms of the Creative Commons Attribution 4.0 International license.

### *In vitro* activities of enzymes potentially involved in NAD^+^ regeneration during axenic growth of S. aciditrophicus with crotonate.

Soluble extracts of S. aciditrophicus grown axenically with crotonate exhibit formate:NAD^+^ and reverse NADH:CO_2_ oxidoreductase activities, along with a membrane-bound formate:DMN (2,5-dimethyl-1,4-naphthoquinone = (M)MK-analogue) oxidoreductase activity (Table 1) (17). However, only soluble H_2_:NAD^+^, but no membrane-bound H_2_:DMN oxidoreductase activities are present, which is line with the lack of genes encoding membrane-bound hydrogenases. Membrane fractions did not exhibit a NADH:DMN oxidoreductase activity, that would have been expected for a respiratory complex I.

**TABLE 1:** *In vitro* enzyme activities in extracts of S. aciditrophicus grown axenically with crotonate^*b*^

Donor → acceptor	Fraction of extracts	Reaction followed	Activity (nmol mg^−1^ min^−1^)
NADH → CO_2_	S	NADH oxidation	20 ± 11/23 ± 6^*a*^
NADH + Fd_red_^–^→ CO_2_	S	Fd_red_^–^ oxidation	<0.1
NADH → DMN	M	NADH oxidation	<0.7
NADH → Enoyl-CoA	S	NADH oxidation	<0.1
NADH → Enoyl-CoA + Fd	S	Fd reduction	<0.1
Fd_red_^–^ → NAD^+^	S	Fd_red_^–^ oxidation	<0.1
Fd_red_^–^ + Acyl-CoA → NAD^+^	S	Fd_red_^–^ oxidation	<0.1
Fd_red_^–^ → Enoyl-CoA	S	Fd_red_^–^ oxidation	<0.1
Fd_red_^–^ → CO_2_	S	Fd_red_^–^ oxidation	<0.1
Formate → NAD^+^	S	NAD^+^ reduction	251 ± 36^*a*^
Formate → NAD^+^ + Fd	S	Fd reduction	<0.1
Formate → TMN	M	TMN reduction	4,200 ± 400^*a*^
H_2_ → NAD^+^	S	NAD^+^ reduction	290 ± 36^*a*^
H_2_ → TMN	M	TMN reduction	<0.5^*a*^
3-OH-butyryl-CoA → NAD^+^	S	NAD^+^ reduction	730 ± 51^*a*^
Pyruvate (+CoA) → Fd	S	Fd reduction	12 ± 1
Pyruvate (+CoA) → Fd → NAD^+^	M	NAD^+^ reduction	18 ± 1

a Values taken from Agne et al. (17).

b As enoyl-CoA substrates, cyclohex-1-ene-1-carboxyl-CoA/cyclohexa-1,5-dienoyl-CoA were used; as substrate for an acyl-CoA DH cyclohex-1-ene-1-carboxyl-CoA was used. S, soluble fraction; M, membrane fraction. For activity measurements in this work, mean value standard deviation are given (*n *≥ 2). Both, TMN and DMN serve as 8-MMK analogues at equal activities.

For testing the involvement of potential Fd-dependent reactions during NAD^+^ regeneration, we purified Fd from soluble S. aciditrophicus cell extracts by anion exchange and size exclusion chromatography. It showed a UV/vis spectrum with a 390:280 nm absorbance ratio of 0.78 (Fig. S2A), which indicates a high purity and [4Fe-4S]-cluster occupation in comparison to the heterologously produced gene product SYN_03059 (390:280 ratio of 0.63) (12). The functional integrity of the purified Fd was demonstrated by its virtual complete reduction in the presence of 5 mM pyruvate, 0.5 mM CoA, and soluble extracts of S. aciditrophicus by the action of pyruvate:Fd oxidoreductase (Fig. S2B). This enzyme is involved in the assimilation of acetyl-CoA formed in the oxidative branch. We then tested the possibility whether the observed NADH:CO_2_ oxidoreductase activity may be assigned to an electron-confurcating FDH, which could drive the reduction of CO_2_ with NADH by the exergonic reduction of CO_2_ with reduced Fd (Fd_red_^–^). Neither in the presence nor absence of NADH, Fd_red_^–^ served as donor for CO_2_ reduction. In the bifurcation direction, no reduction of Fd was observed in the presence of formate/H_2_ and NAD^+^. Both results argue against the presence of electron-confurcating FDHs or hydrogenases, which is in full agreement with the amino acid sequence analyses of the NADH/FMN-binding domains in previous work (12) and in this work (Fig. S1).

10.1128/mbio.03740-21.3 FIG S2 Absorption spectrum and reduction of Fd from S. aciditrophicus. (A) Ultra-violet/visible spectrum of Fd from S. aciditrophicus as isolated from wild type cells with absorption maxima at 280 and 379 nm. (B) Reduction of S. aciditrophicus Fd with 5 mM pyruvate, 0.5 mM CoA, and soluble cell extract of S. aciditrophicus at 37°C. Spectra were recorded in the direction of the arrow, at time points before 1, 2, 5, and 15 min after the addition of CoA. Download FIG S2, DOCX file, 0.1 MB. Copyright © 2022 Agne et al. 2022 Agne et al. https://creativecommons.org/licenses/by/4.0/ This content is distributed under the terms of the Creative Commons Attribution 4.0 International license.

We further tested the possibility whether NADH is oxidized by cyclohex-1-ene-1-carboxyl-CoA or cyclohex-1,5-diene-1-carboxyl-CoA, the enoyl-CoA substrates of two acyl-CoA DHs involved in the reductive branch of crotonate degradation (Fig. 2). No such activity was observed, and Fd added to such assays was not reduced (Fig. 2). This observation is in full accordance with previous studies (17) and rules out that an electron-bifurcating ETF is involved in mediating electron transfer from NADH to acyl-CoA DHs and Fd.

We finally tested the presence of a membrane-bound Fd_red_^–^:NAD^+^ oxidoreductase activity in membranes of cells grown axenically with crotonate, a reaction typically catalyzed by Rnf complexes (35). For this purpose, continuous Fd reduction was accomplished in the presence of 5 mM pyruvate, 0.5 mM CoA, and soluble extracts containing Fd and pyruvate:Fd oxidoreductase activity (Table 1). Notably, the assays contained >10 mM Na^+^ ions that may be required for Rnf activity (36). Using this setup, the membrane fraction of S. aciditrophicus catalyzed the pyruvate and CoA dependent reduction of NAD^+^, which indicates that Rnf is active during syntrophic growth with crotonate (Table 1). We propose that the reverse reaction, driven by an ion motive force, is crucial for providing Fd_red_^–^ for acetyl-CoA assimilation.

### Hypophosphite is a strong inhibitor of axenic growth of S. aciditrophicus with crotonate and of *in vitro* mFDH activity.

**FIG 3:**
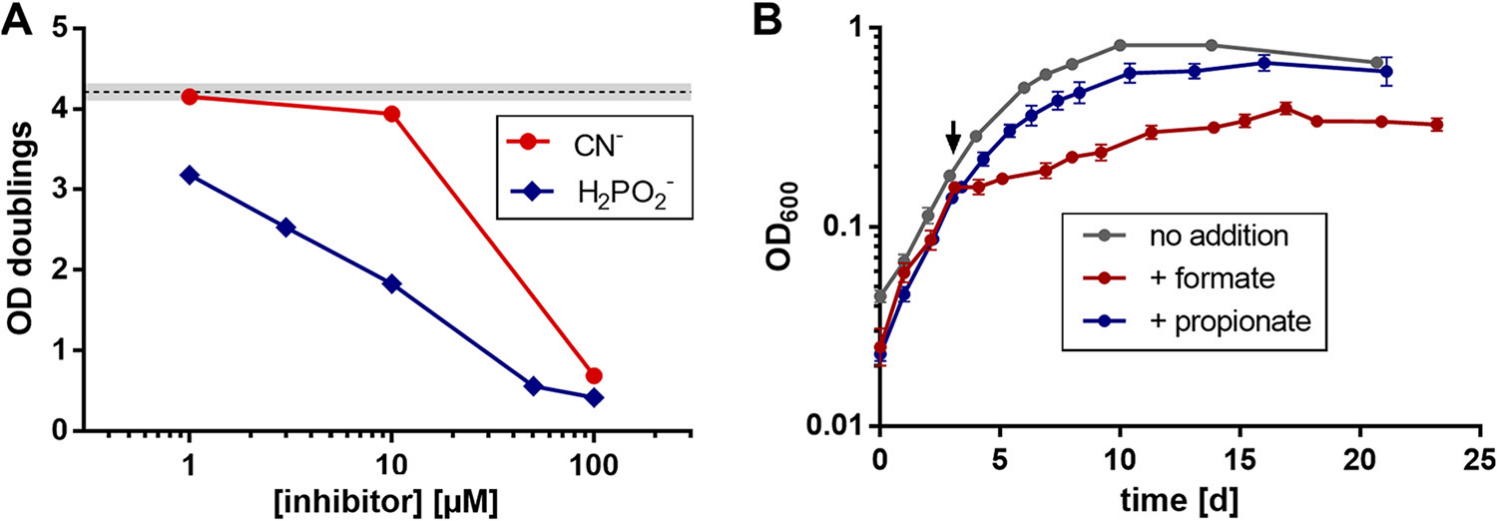
Growth inhibition of S. aciditrophicus cultures. (A) Effect of inhibitor concentrations on the maximum number of doubling events during axenic growth of S. aciditrophicus cultures with crotonate for 10 days; • cyanide, ♦ hypophosphite. The dashed line marks the average doubling events of cultures in the absence of inhibitors, the gray shading marks the SD (*n* = 3). (B) Effect of the addition of formate and propionate on the growth phenotype of S. aciditrophicus cultures. The arrow indicates the time point when the acids were added to the growing cultures. OD values are given as means ± SD (*n* = 4 for formate, *n* = 3 for no addition and propionate).

To further evaluate the role of FDHs and hydrogenases during axenic crotonate degradation, the effect of the FDH-specific inhibitor hypophosphite (H_2_PO_2_^–^, a substrate analogue of formate) (37), and the typical hydrogenase inhibitor cyanide on axenic growth was tested. The presence of 1 μM hypophosphite resulted in a significant negative effect on growth as documented by the maximal number of doublings reached within 10 days (Fig. 3A); at 100 μM hypophosphite, growth was almost completely abolished. In contrast, the hydrogenase-specific inhibitor cyanide exhibited only at very high concentrations (100 μM) an effect on growth. This result confirms that FDHs rather than hydrogenases play a crucial role during axenic crotonate degradation. We further tested the effect of hypophosphite on the *in vitro* activities of sFDH in soluble extracts and mFDH in washed membranes. It effectively inhibited mFDH activity using 2,3,5-trimethyl-1,4-naphthoquinone (TMN, (M)MK analogue) as acceptor with a 50% inhibition observed at 30 μM (Fig. S3). In contrast, NAD^+^-dependent sFDH activity showed only a very low susceptibility with 50% inhibition at an around 1,000-fold higher hypophosphite concentration. These results indicate that hypophosphite acts as a specific inhibitor of mFDH in S. aciditrophicus, which provides a rational for its inhibitory effect on axenic growth with crotonate.

10.1128/mbio.03740-21.4 FIG S3 Inhibition of FDH activities by hypophosphite. Effect of increasing hyophosphite concentrations on *in vitro* TMN- (mFDH) or NAD^+^-dependent (sFDH) activities in washed membranes or soluble cell extract, respectively. 100% activity refers to 1.14 U g^−1^ cell wet weight for TMN dependent mFDH activity and 1.55 U g^−1^ cell wet weight for NAD^+^ dependent sFDH activity. Download FIG S3, DOCX file, 0.05 MB. Copyright © 2022 Agne et al. 2022 Agne et al. https://creativecommons.org/licenses/by/4.0/ This content is distributed under the terms of the Creative Commons Attribution 4.0 International license.

### Cellular reduction potentials of redox couples allow for an electron transfer from NADH to CO_2_.

Our observations indicate that mFDH is crucial for axenic growth of S. aciditrophicus with crotonate suggesting that formate cycling connects the oxidative and reductive branches of crotonate catabolism. In such a process, the formate formed by a cytoplasmic NAD^+^ regenerating sFDH is transported to the periplasm via a bidirectional FocA-like channel (17, 38), and serves as electron donor for enoyl-CoA reduction involving mFDH, 8-MMK, EMO, ETF, and acyl-CoA DH. Electron transfer from 3-hydroxybutyryl-CoA (E°’ = −250 mV) (6) via NAD^+^/NADH (E°’ = −320 mV) to CO_2_ (E°’ = −430 mV for the CO_2_/formate couple) is clearly endergonic under standard conditions (ΔG°’ = +34.8 kJ mol**^−^**^1^) but becomes possible when a methanogenic partner captures the formate keeping concentrations in the low double-digit μM range (6).

To investigate whether the NADH-dependent CO_2_ reduction is also thermodynamically feasible in axenically grown cells, we determined the CO_2_/formate and NAD^+^/NADH ratios during growth of S. aciditrophicus with crotonate. Extracellular formate concentrations of an exponentially growing culture were determined by ion chromatography, and were on average 21 ± 9 μM (mean of 10 measurements ± SD), and in the range where syntrophic electron transfer via formate occurs (6). Taking into account 20% CO_2_ (vol/vol) in the gaseous phase, and assuming that intra- and extracellular CO_2_ and formate can equilibrate via the bidirectional FocA channel (38), the *in situ* reduction potential of CO_2_/formate was −298 ± 5 mV. The determination of cellular NAD^+^/NADH ratios was accomplished by adopting an MS-based metabolite determination method developed previously (39) to S. aciditrophicus. Briefly, it involves an acidic extraction step during which NAD^+^ is stable but NADH spontaneously hydrolyses to adenosine 5′-diphosphoribose (ADP-ribose) and nicotinamide. The cellular NADH concentration was therefore deduced from the measured concentration of ADP-ribose (for details see Materials and Methods). Using this method, the NADH/NAD^+^ ratios were first determined for aerobically grown E. coli as a control. The ratio of 0.2 determined fitted well to previously reported values that are in a range from 0.03 (39) to 0.3 (40). For S. aciditrophicus, we determined a NADH/NAD^+^ ratio of 0.28 ± 0.04 (mean of four independent experiments ± SD). This results in a cellular reduction potential of −304 ± 2 mV, that is slightly below that of the CO_2_/formate couple. In summary, the reduction potentials deduced from the NAD^+^/NADH and formate/CO_2_ ratios indicate that NADH-dependent CO_2_ reduction under axenic growth conditions is thermodynamically just as possible as during syntrophic conversion of fatty acids into methane (4, 41).

### Axenic growth of S. aciditrophicus with crotonate is susceptible to extracellular formate concentrations and alters the product stoichiometry.

**FIG 4:**
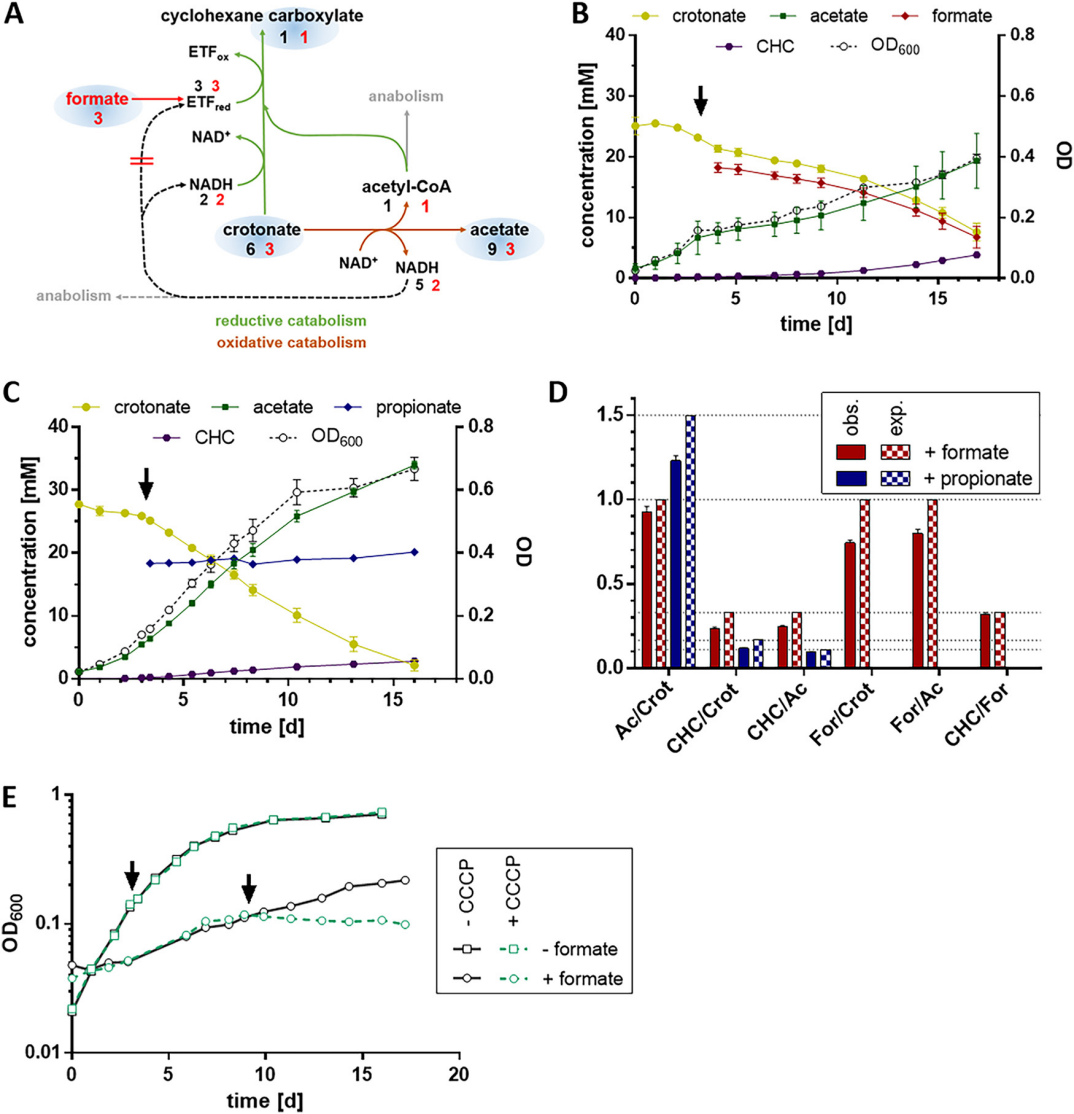
Effect of extracellular formate on axenic growth of S. aciditrophicus with crotonate. (A) Simplified scheme of S. aciditrophicus crotonate degradation pathway highlighting the stoichiometry of metabolites in the presence (red numbers) or absence (black numbers) of externally added formate. Green arrows depict the reductive catabolic branch; brown arrows depict the oxidative catabolic branch. Free acids that were quantified in the supernatant are shown with blue shading. (B, C) Free acid concentrations in the culture supernatant and OD_600_ of S. aciditrophicus batch cultures. At day 3, 20 mM sodium formate (B) or 20 mM sodium propionate (C) was added (arrows). All data points except for propionate represent mean values ± SEM (*n* ≥ 3). (D) Ratios of substrate and product carboxylic acids during axenic growth with 30 mM crotonate + 20 mM propionate (blue bars) and in the presence of 30 mM crotonate + 20 mM formate (red bars). Calculated values according to (A) are presented in checkered bars, measured values are shown in filled bars. Mean values ± standard error are given for 70 data points from seven biological replicates (red bars) or 38 data points from four biological replicates (blue bars). Ac, acetate; Crot, crotonate; CHC, cyclohexane carboxylate; For , formate. (E) Growth curves of S. aciditrophicus cultures in the presence or absence of formate. At the indicated time points, 40 μM CCCP was added.

A major prerequisite for formate cycling is that the external formate concentration is kept at a low level (≈20 μM). For example, already at 1 mM formate, the reduction potential of CO_2_/formate drops to −350 mV, making CO_2_ reduction by NADH endergonic (≈ +9 kJ mol**^−^**^1^). On the other side, external formate at high concentrations may substitute for the NADH formed in the oxidative branch as alternative electron donor for enoyl-CoA reduction. In such a scenario, carbon flux through the oxidative branch should be largely diminished (Fig. 2). When 20 mM formate was added to an exponentially growing culture at around OD 0.2, growth immediately halted, and then continued at a lower rate (Fig. 3B). In control experiments, the addition of propionate (20 mM), that plays no role during crotonate degradation, had no noticeable effect on the growth curve, which rules out that the effect caused by formate is due to a change of pH. Continuous control of pH during the entire growth experiment confirmed this conclusion. The formate added in such experiments was immediately consumed, whereas in the control propionate concentration remained constant (Fig. 4B, C). If indeed formate substitutes for NADH for enoyl-CoA reduction, the substrate/product ratios should be markedly effected (Fig. 4A). For example, the ratio of acetate formed per crotonate consumed should be shifted from 1.5 to 1.0, that of cyclohexanecarboxylate formed per crotonate consumed from 1:6 to 1:3; the cyclohexanecarboxylate to acetate ratio is expected to change from 1:9 to 1:3 (Fig. 4A). Overall, the measured ratios match the theoretical expectations very well (Fig. 4AD). The generally slightly smaller values observed than theoretically predicted reflect the use of acetyl-CoA and NADH formed in the oxidative branch for assimilatory processes (26). The results indicate that high external formate concentrations switch axenic crotonate degradation further into the direction of a respiratory mode of energy metabolism. Under these conditions, the oxidative branch is only used for balancing the two NADH-dependent reactions of the reductive branch (Fig. 2), and for providing reducing equivalents for acetyl-CoA assimilation via Rnf.

To further confirm the suggested formate-induced shift toward a higher contribution of ETP versus SLP, we tested the effect of 40 μM carbonyl cyanide *m*-chlorophenyl hydrazone (CCCP) (Fig. 4E). No significant impact on growth with crotonate was observed suggesting that this CCCP concentration was too low to affect the exponentially growing cells. In contrast, a complete abortion of growth occurred in the presence of 20 mM formate. This higher susceptibility toward an uncoupler is in line with the anticipated shift toward ETP.

## DISCUSSION

The results obtained in this work, together with supportive previous observations, provide multiple lines of evidence that the oxidative and reductive branches of unsaturated scFA fermentation in S. aciditrophicus are linked by formate cycling involving an energy conserving redox loop: (i) the previous reconstitution of the electron transfer chain from formate to enoyl-CoA via a redox-loop in both axenically and syntrophically grown cells (17); (ii) the presence of NADH- and DMN/TMN-dependent FDH activities in cell extracts grown with crotonate; (iii) the lack of gene products and their corresponding activities that could be involved in alternative NAD^+^-regeneration pathways such as NADH:quinone oxidoreductases, or electron-bifurcating ETFs/FDHs/hydrogenases; (iv) the inhibition of axenic growth with crotonate and mFDH activity by the formate analogue hypophosphite; (v) the effect of external formate on growth with crotonate; (vi) the altered stoichiometries between substrates used/products formed in the presence of external formate; and (vii) the cellular NAD^+^/NADH and extracellular CO_2_/formate ratios that are in agreement with the proposed electron transfer events.

**FIG 5:**
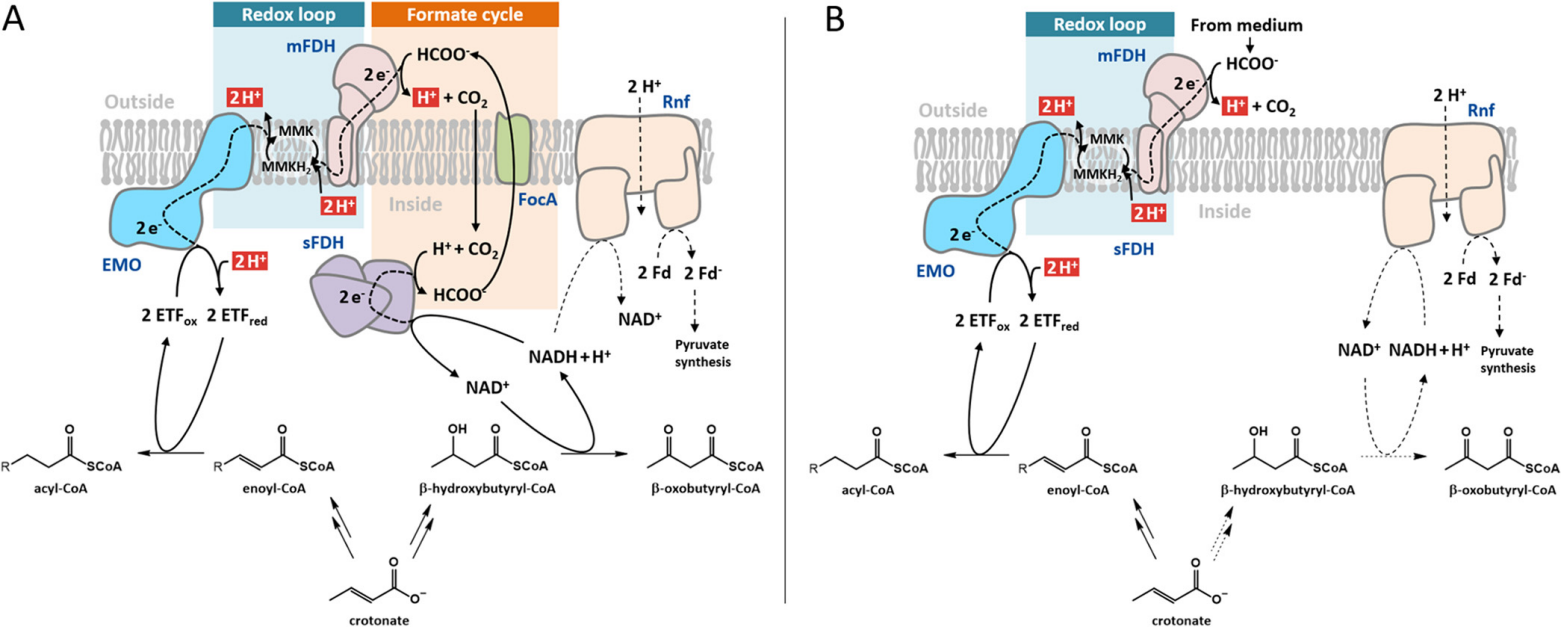
Electron transfer components/processes involved during axenic crotonate degradation in S. aciditrophicus. (A) At low formate concentrations ≈ 20 μM the NADH formed in the oxidative branch by 3-hydroxybutyryl-CoA DH is used to form ETF_red_ as donor for acyl-CoA DH in the reductive branch via formate cycling and the redox loop. (B) At formate concentrations ≥1 mM, intracellular formate formation is abolished, and external formate serves as donor for enoyl-CoA reduction via the redox loop. The oxidative branch is predominantly used for providing reducing equivalents and acetyl-CoA for pyruvate synthesis via Rnf. In addition, NADH/acetyl-CoA formed in the oxidative branch is used for chain elongation in the reductive branch (not shown here, see Fig. 2).

During formate cycling, the NADH formed by 3-hydroxyacyl-CoA DH is used for intracellular reduction of CO_2_ to formate, which, after transport via a FocA-like channel to the periplasm (gene locus SYN_RS11905), is re-oxidized by 8-MMK catalyzed by mFDH (Fig. 5A). Electron transfer from the externally orientated mFDH to internally orientated EMO forms a redox loop that in total results in the transport of four protons during the reduction of enoyl-CoA by formate. The Fd_red_^–^ required for acetyl-CoA assimilation via pyruvate synthase is generated from NADH by Rnf at the expense of an ion motive force. The model does not include the possibility that a significant amount of NAD^+^ may be regenerated via the recently characterized non-bifurcating NADH-dependent [Fe-Fe]-hydrogenase (12). In accordance, the experimentally determined electron balances during axenic growth with crotonate rule out a significant electron loss via H_2,_ and there are no membrane-bound uptake hydrogenases in S. aciditrophicus (15). However, in natural environments, where H_2_-consuming organisms are present, NAD^+^ regeneration via this hydrogenase may become important.

At the high extracellular formate concentrations used in a part of our growth experiments, NADH-dependent CO_2_ reduction cannot occur. Here, crotonate degradation proceeds via the following equation (ΔG°’ was calculated from ΔG°_f_ listed by Thauer et al. (21); ΔG°_f_ value for cyclohexanecarboxylate was taken from Moutakki et al. [26]):

$${\text{3 crotonate}}^{–}+{\text{ 3 formate}}^{–}+{\text{ 2 H}}_{\text{2}}\text{O}\to {\text{3 acetate}}^{–}+{\text{ cyclohexanecarboxylate}}^{–}+{\text{ 2 HCO}}_{\text{3}}{}^{–}$$

ΔG°’ = –67.8 kJ per mol crotonate

Here, external formate serves as only electron donor for the three enoyl-CoA reduction steps via the redox loop as indicated by the continuous consumption of externally added formate (Fig. 5B). The NAD^+^ reduced during the oxidation of crotonyl-CoA to acetyl-CoA may be regenerated by Rnf-dependent Fd reduction for anabolic purposes, and by the two NADH-dependent reduction steps in the reductive branch (Fig. 2).

The redox loop will substantially contribute to energy conservation during axenic growth of S. aciditrophicus with crotonate because for the six crotonate converted into nine acetate and one cyclohexanecarboxylate, 12 protons (four for each acyl-CoA DH reaction, Fig. 2) are transported across the cytoplasmic membrane giving two protons translocated per crotonate. Assuming that ATP synthase translocates three to four protons per ATP synthesized, the overall ATP yield will on average double from 0.6 to 1.1-1.26 ATP (+0.5-0.66 via ETP) per crotonate. This range fits well to the molar growth yield and is in the range of the theoretically estimated ΔG value of −48 kJ per mol crotonate as determined from the ΔG°_f_ values (26). The diminished growth rate in the presence of external formate cannot be explained by the energy yield, which is theoretically even higher than without formate. One likely explanation is that formate is imported into the cytoplasm via the bidirectional FocA channel (38), where it serves as a donor for NAD^+^ reduction by sFDH. Such a reaction would substantially increase the cellular NADH/NAD^+^ ratio and in turn impair NAD^+^-dependent 3-hydroxybutyryl-CoA DH, a thermodynamically limiting reaction in the oxidative branch.

Under environmental conditions where low formate concentrations will prevail, three out of five NAD^+^ are regenerated via a membrane-potential generating redox-loop (Fig. 2). For this reason, we propose that axenic growth with unsaturated scFA of secondary fermenting bacteria should no longer be referred to as a fermentative, but rather as a respiratory process. Because enoyl-CoA compounds serve as acceptors for terminal acyl-CoA DH, we term this process enoyl-CoA respiration as a novel mode of respiratory metabolism. Though cyclohexanecarboxylate formation during axenic crotonate degradation in S. aciditrophicus appears to represent a rather specialized solution to regenerate the reducing equivalents formed in the oxidative branch, it is very likely that enoyl-CoA respiration is abundant in syntrophic bacteria that axenically grow with unsaturated scFA. As an example, Syntrophomonas wolfei forms two acetate and one butyrate from two crotonate according to the pathway depicted in Fig. 1 (42). Proteomic studies clearly showed that, similar as in S. aciditrophicus, the characteristic redox-loop oxidoreductase EMO is non-differentially produced during syntrophic growth with butyrate and axenic growth with crotonate (13, 14). Moreover, under such growth conditions, mFDH and membrane-bound hydrogenase activities have been detected, supported by proteomic studies (5, 13, 19). Most likely, cycling of H_2_ instead of formate is involved in the proposed crotonyl-CoA respiration in S. wolfei. As a further alternative, a NADH:(M)MK oxidoreductase could be involved, which would represent an enoyl-CoA respiration variant without the involvement of a formate/hydrogen loop (43). Though these examples indicate that variations concerning the path of electrons from NADH to the (M)MK pool may exist, they support the concept of EMO/(M)MK dependent enoyl-CoA respiration in syntrophs growing with unsaturated fatty acids. This mode of energy conservation during crotonate degradation fundamentally differs from that in Firmicutes. In the latter, regeneration of NADH formed in the oxidative branch is regenerated by a butyryl-CoA DH/ETF complex that may couple the endergonic reduction of Fd by NADH to exergonic crotonyl-CoA reduction by NADH via flavin-based electron bifurcation (22, 44). The redox-loop dependent enoyl-CoA respiration identified in this work also clearly differs from hydrogen-dependent caffeate respiration in *Acetobacterium woodi*, where a NADH-dependent, electron-bifurcating caffeyl-CoA reductase/Fd-ETF complex is involved (45). In this organism, the reoxidation of Fd_red_^–^ with NAD^+^ by an Rnf-complex then translocates sodium-ions outside the cell (46).

The energy-conserving intracellular formation of low-potential redox carriers that are reoxidized via periplasmic oxidoreductases, quinones, and intracellular high-potential acceptors has originally been described for hydrogen cycling in sulfate-reducing bacteria (SRB) (47). Meanwhile, evidence has also been gained for formate cycling in SRB (48) though both, hydrogen and/or formate cycling, appear not to be essential for all SRBs (49). Hydrogen cycling involved in energy conservation has also been demonstrated in *Methanosarcina* species during methane formation from acetate or methanol (50, 51), and is proposed to play a role in the metal-respiring Geobacter sulfurreducens (52). Recently, an intracellular variant of hydrogen cycling has been described in the acetogenic *Acetobacterium woodi*, where during the oxidation of fructose to three acetate H_2_ is produced that serves as electron donor for the cytoplasmic reduction of CO_2_ to acetate (53).

The establishment of formate and/or hydrogen cycling coupled to enoyl-CoA respiration allows syntrophic bacteria to substantially increase the energy yield via ETP in the absence of a methanogenic partner. It depends on efficient uptake mFDHs (or hydrogenases) to keep the external formate (H_2_) concentration low, which is mandatory for the cycling process. Indeed, formate remained constantly at ≈20 μM during exponential growth, which represents only 0.1% of the initially added crotonate, and which is close to formate concentrations found during syntrophic formation of methane from crotonate (6). This finding demonstrates an efficient capture of formate by the externally orientated mFDH. The question arises whether the mandatory interaction between a syntrophic bacterium and a methanogenic archaeon during growth with saturated scFA turns into a competition for formate during growth with an unsaturated scFA. This question applies for all types of syntrophic interactions where formate/hydrogen cycling represents an alternative to interspecies electron transfer. The answer is probably that in natural environments, a mix of saturated and unsaturated scFA together with other primary fermentation products serve as substrates for syntrophic consortia. Thus, the competition for formate/hydrogen might only play a minor role in comparison to the mandatory establishment of a syntrophic association for the conversion of saturated fatty acids into methane. The observed possibility to capture both internally formed but also externally added formate by the bacterial partner may allow for a higher flexibility in adjusting electron transfer balances during the establishment and maintenance of stable syntrophic associations with methanogenic partners. In this context, it is of great advantage that S. aciditrophicus uses the same enzyme inventory during syntrophic and axenic growth (31), which circumvents a costly *de novo* synthesis of proteins during frequent fluctuations of carbons sources.

## MATERIALS AND METHODS

### Chemicals.

The chemicals used were of analytic grade and were purchased from Fluka (Buchs, Switzerland), Merck (Darmstadt, Germany), Roth (Karlsruhe, Germany), Sigma-Aldrich (St. Louis, MO), AppliChem (Darmstadt, Germany), and Bio-Rad (Hercules, CA). According to the procedure of Jacobsen et al. (54), 2,3,-Dimethyl-1,4-naphthoquinone (DMN) was synthesized, and 2,3,5-trimethyl-1,4-naphthoquinone (TMN) according to the method of Schmid et al. (55).

### Cultivation of bacteria.

S. aciditrophicus strain SB (DSM 26646) was obtained from Michael J. McInerney (Norman, OK) and cultivated anaerobically as described before in mineral salt medium (pH 7.3) with crotonate (30 mM) as the sole carbon source and energy source without addition of rumen fluid (56).

Flasks for growth curves were inoculated with 10 mL from a exponentially growing culture which was transferred to fresh medium at the same ratio every 9 to 10 days. When the inhibitors hypophosphite (1, 3, 10, 50, and 100 mM final concentration) or cyanide (1, 10, and 100 mM final concentration) were used, they were added to the growth medium before inoculation. Formate (20 mM), propionate (20 mM), or the uncoupling agent carbonylcyanid-m-chlorphenylhydrazon (CCCP, 40 μM) were added on day 3 after inoculation to cultures growing with crotonate, or on day 9 after inoculation to cultures growing on crotonate + formate. Additions were made from anaerobic sterile stock solutions by diluting them at least 1:50. Samples were withdrawn with a syringe and optical density was measured at 600 nm with an Ultrospec 1100 pro spectrophotometer (Amersham Bioscience, Little Chalfont, UK) after 10 min incubation on air when the redox indicator resazurin was completely oxidized. Directly before measurement, the samples were shortly agitated to reverse cell sedimentation. Afterwards, samples were stored at −20°C for further use.

Cell mass was obtained by anaerobic cultivation in the 200 L scale with the medium described above and harvested at the late exponential growth phase (OD_600_ ≈ 1). Until further usage, cell material was stored in liquid nitrogen.

### Cell fractionation.

For cell fractionation, 10 g cells were suspended in 20 mL TB buffer (20 mM Tris-HCl, 5 mM MgCl_2_, pH 7.8) plus spatula tips of DNase I, lysozyme as well as dithioerythritol, and were opened by a passage through a French pressure cell (American Instrument Company, Hartland, WI) at 1,100 psi. Intact cells were sedimented by a centrifugation at 25,000 × *g* (20 min). Soluble cell extract and crude membranes were separated by a centrifugation at 200,000 × *g* (1 h). Membranes were washed by homogenizing them in 20-mL extraction buffer followed by another centrifugation at 200,000 × *g* (1 h). Washed membranes were homogenized in 5-mL TB buffer. All steps were performed anaerobically at 4 °C to 8°C.

### Enrichment of Fd.

Fd was enriched from the 200,000 × *g* supernatant obtained from 10 g of S. aciditrophicus grown axenically with crotonate. Then, 20 mL of the supernatant were applied to a 15-mL DEAE Sepharose column that had been equilibrated with 20 mM Triethanolamine hydrochloride (TEA), 10% glycerol (wt/vol) (buffer A) containing 100 mM NaCl. After washing for one column volume (CV), a gradient from 100 to 500 mM NaCl in buffer A over three CV was applied. The brownish band containing Fd was concentrated to 1 mL and applied to a 320-mL HiLoad 26/600 Superdex 200 pg (Cytiva, Marlborough, MA) gel filtration column that had been equilibrated with buffer A containing 150 mM KCl with a flow rate of 2 mL min^−1^. Fd eluted in 12 mL and was concentrated to 0.5 ml using Vivaspin Turbo 4 concentrators (10,000 MWCO, Pall Corporation, New York, USA).

### Determination of enzyme activities/inhibition assays.

With the exception of hydrogenase, all electron transfer processes were analyzed in an N_2_/H_2_ (95:5 by vol.) atmosphere at 30°C in MOPS buffer (25 mM, pH 7.3) unless otherwise stated.

For determination of FDH activities, 10 μL of S. aciditrophicus soluble cell extract or washed membranes were diluted in 430-μL MOPS buffer containing either 90 μM DMN/TMN, 2 mM NAD^+^, or 1 mM NAD^+^ plus 3 μM Fd, respectively. For inhibition studies, 50-μL stock solutions of sodium cyanide or sodium hypophosphite in different concentrations were added and the cuvettes were closed with rubber stoppers. As soon as the absorption was stable, the reaction was started by the addition of 2 mM sodium formate by a syringe and followed at 272 nm, 340 nm, or 410 nm using the following absorption coefficients: DMN, ε_272_ = 16 mM**^−^**^1 ^cm**^−^**^1^; NADH, ε_340_ = 6.25 mM**^−^**^1 ^cm**^−^**^1^; Fd, ε_410_ = 37 mM**^−^**^1 ^cm**^−^**^1^).

CO_2_ dependent NADH, Fd _red_-, or NADH plus Fd_red_- oxidation was followed by the decrease of NADH absorption at 340 nm (ε_340_ = 6.25 mM^−1 ^cm^–^1) or the increase of Fd absorption at 410 nm (ε_410_ = 37 mM^−1 ^cm^−1^). The reaction mixture contained 0.1 to 1 mg ml^−1^ S. aciditrophicus soluble cell extract and 0.25 mM NADH in a total volume of 120-μL potassium phosphate buffer (250 mM, pH 6.5). The assay was performed in stoppered glass cuvettes and the reaction was started by the addition of 200 μl N_2_/CO_2_ (80:20) gas using a syringe.

NAD^+^ or TMN dependent hydrogenase activities were determined spectroscopically using stoppered glass cuvettes under a N_2_ atmosphere. For this purpose, 10 to 20 μL of *S*. aciditrophicus soluble cell extract or washed membranes were diluted in 450 μL MOPS buffer containing 1 mM NAD^+^ or 90 μM DMN, respectively. The reaction was started by the addition of 200 μL H_2_/CO_2_ (80:20) by a syringe and followed at 340 or 272 nm.

NAD^+^ and NAD^+^ plus acyl-CoA dependent Fd_red_- oxidation was determined using 0.1 mg ml^−1^ S. aciditrophicus soluble cell extract, 3 μM Fd_red_-, and 0.5 mM cyclohex-1-ene-1-carboxyl-CoA in 120-μL MOPS buffer. The reaction was started with 0.5 mM NAD^+^ and followed at 410 nm.

Enoyl-CoA dependent Fd_red_- oxidation was followed in the presence of 0.1 mg mL^−1^ S. aciditrophicus soluble cell extract and 3 μM Fd_red_- in 120-μL MOPS buffer. The reaction was started with 0.5 mM cyclohex-1-ene-1-carboxyl-CoA or cyclohexa-1,5-dienoyl-CoA and followed at 410 nm.

DMN, enoyl-CoA, or enoyl-CoA plus Fd dependent NADH oxidation was followed by the decrease of NADH or Fd absorption at 340 or 410 nm, respectively. The reaction mixture contained 0.1 to 1 mg mL^−1^ S. aciditrophicus soluble cell extract, 1 mM NADH, and 3 μM Fd (for Enoyl-CoA/Fd dependent reaction) in 120-μL MOPS buffer, and was started with wither 90 μM DMN, 0.5 mM cyclohex-1-ene-1-carboxyl-CoA, or cyclohexa-1,5-dienoyl-CoA (NADH, ε_340_ = 6.25 mM^−1 ^cm^−1^; Fd, ε_410_ = 37 mM^−1 ^cm^−1^).

Pyruvate and CoA dependent Fd reduction was determined with 0.1 mg mL^−1^ S. aciditrophicus soluble cell extract, 3 μM Fd, and 5 mM sodium pyruvate in 120-μM MOPS buffer. The reaction was started with 0.5 mM CoA and followed at 390 nm (ε_390_ = 40 mM^−1 ^cm^−1^).

Rnf activities were determined spectroscopically, following the increase of NADH absorption at 340 nm (ε_340_ = 6.25 mM^−1 ^cm^–^1). The reaction mixture contained 0.15 mg mL^−1^ S. aciditrophicus washed membrane, 0.1 mg mL^−1^ desalted soluble cell extract, 5 mM sodium pyruvate, and 0.5 mM CoA in TB buffer with NaCl (20 mM Tris-HCl, 5 mM MgCl_2_, 10 mM NaCl, pH 7.8). The reaction was started by addition of 1 mM NAD^+^. Control assays without the addition of pyruvate were performed; the background NAD^+^ reduction activity was subtracted as baseline from the measured activities.

### Quantification of carboxylic acids in culture supernatants.

For the quantification of substrates and growth products, frozen culture samples were thawed, and cells were sedimented by centrifugation at 2,500 × *g* (5 min). Then, 50 μL of the supernatant were mixed with 100 μL MeOH and centrifuged at 15,000 × *g* (10 min, **4°C**) to precipitate residual proteins. Centrifugation was repeated after diluting 21.4 μL supernatant with 300 μL dH_2_O. Next, 10 μL of the final supernatant were analyzed by a ion chromatography system (ICS-2100, Thermo Fisher Scientific, Waltham, MA) equipped with an Ion-Pac AS11-HC anion exchanger column (Thermo Fisher Scientific). Analyte separation was achieved by sequential elution with a KOH gradient starting at 0 mM for 1 min, followed by 2 mM for 7 min and increasing to 25 mM within 6 min, followed by a washing step at 60 mM for 2.5 min and an equilibration step at 0 mM for 4.5 min (flow rate 0.38 ml min**^−^**^1^, column temperature 30°C).

### Calculation of the fermentation balance.

To account for divergences due to sample preparation and measurement, chloride ions were used as internal standard for the calculation of substrate and/or growth product ratios. Thus, analyte peak areas were normalized to the peak area of chloride before converting them to concentrations according to calibration curves recorded with the respective authentic standard. Ratios of different acids were calculated from the differences of the respective acids between concentrations of samples taken at the same time points. All ratios of every possible combination of time points from one growth curve were used to calculate a mean value after the elimination of outliers by a ROUT test (Q = 1%) with GraphPad Prism 6 (GraphPad Software, La Jolla, CA). The standard error of the mean (SEM) was calculated by dividing the standard deviation of the data set by the square root of the number of samples taken. The mean ratios of several growth curves were combined to obtain an overall mean and its SEM was calculated by Gaussian error propagation.

### Determination of cellular NAD(H) and CoA ester concentrations.

To determine the concentration of NAD^+^, NADH, and CoA esters in living cells, S. aciditrophicus cultures were harvested by rapid filtration during exponential growth at an OD_600_ of approximately 0.3. Therefore, a 10-mL cell culture was applied onto a regenerated cellulose filter (0.2 μm pore size, Sartorius, Göttingen, Germany) and washed two times with 20 mL of 37°C phosphate buffer (5 mM KH_2_PO_4_, 5 mM NaCl, pH 7.1). As soon as all the liquid had been passed through, the filter was immediately put into 8 mL precooled quenching solution (acetonitrile/methanol/water [60:20:20] with 15 mM HCl, −20°C). Different amounts of NAD(H) and CoA ester were spiked in to obtain a calibration curve within the sample matrix. For removal of cell debris from the filter, the samples were sonicated in a water bath sonicator (Elmasonic S80, Elma Schmidbauer, Singen, Germany) for 1 min. After 40 min of incubation on ice, the organic solvents were removed by a rotary evaporator (Rotavapor R-114, Büchi Labortechnik, Flavil, Switzerland) at 100 mbar and 40°C for 5 min. The residual liquid was deep frozen in liquid N_2_ and freeze-dried (Alpha 2–4 LD plus, Martin Christ GmbH, Osterode, Germany) over night.

When all liquid was evaporated, the samples were suspended in 200 μL ammonium acetate (10 mM) and centrifuged twice (15,000 *g*, 10 min, 4°C). Then, 8 μL of the supernatant were applied to an Acquity I-class UPLC system (Waters, Milford, MA) equipped with a HSS T3 1.8 μm C_18_ reverse phase column (100 × 2.1 mm, Waters) and coupled to a Synapt G2-Si ESI/Q-TOF mass spectrometer (Waters).

For the separation of ribosyl-ADP and NAD, 1% acetonitrile/0.1% formic acid in water/0.1% formic acid was applied at a flow rate of 0.3 mL min^−1^ for 2 min. Then the acetonitrile/0.1% formic acid concentration increased linearly to 10% within 3 min, followed by a washing step with 80% acetonitrile/0.1% formic acid. For the separation of CoA esters, 5% acetonitrile in ammonium acetate solution (10 mM) was applied at a flow rate of 0.35 mL min^−1^ for 3 min. Then the acetonitrile concentration increased linearly to 20% within 1.5 min, followed by a washing step with 30% acetonitrile. In both cases the mass spectrometer was operated in the Resolution mode with positive polarity and a capillary voltage of 3 kV, a cone voltage of 40 V, 80 V source offset, 150°C source temperature, 450°C desolvation temperature, 1,000 L min^−1^ desolvation gas flow (N_2_), 100 L min^−1^ cone gas flow (N_2_), as well as 6 bar nebulizer pressure.

The areas under the peaks within the chromatogram extracted for the corresponding mass ± 0.02 Da were plotted against the amount of added NAD(H) or CoA ester. The amount of substance per dry weight was then calculated by the following formula:

(1) $$\frac{n}{\mathrm{dry}\;\mathrm{weight}}\;=\frac{\;\frac{A_{0}}{s}}{\alpha \;OD_{600}\;V\;}$$

with *A_0_* representing the *y* axis intercept and *s* the slope of the linear regression line of the chart; *α* is the conversion factor between OD_600_ and dry weight per volume (0.37 g L^−1^ for S. aciditrophicus and 0.46 g L^−1^ for E. coli (57); *V* is the volume of the culture applied to filtration.

### Data availability.

Data are included in the manuscript and in the supplemental material.
