# Supplementary material for: Enoyl-Coenzyme A Respiration via Formate Cycling in Syntrophic Bacteria
Source: mBio. 2022 Feb 1;13(1):e03740-21. doi: 10.1128/mbio.03740-21 (PMC8805022; doi:10.1128/mbio.03740-21)
Supplement: FIG S1 [file mbio.03740-21-sf001.docx]

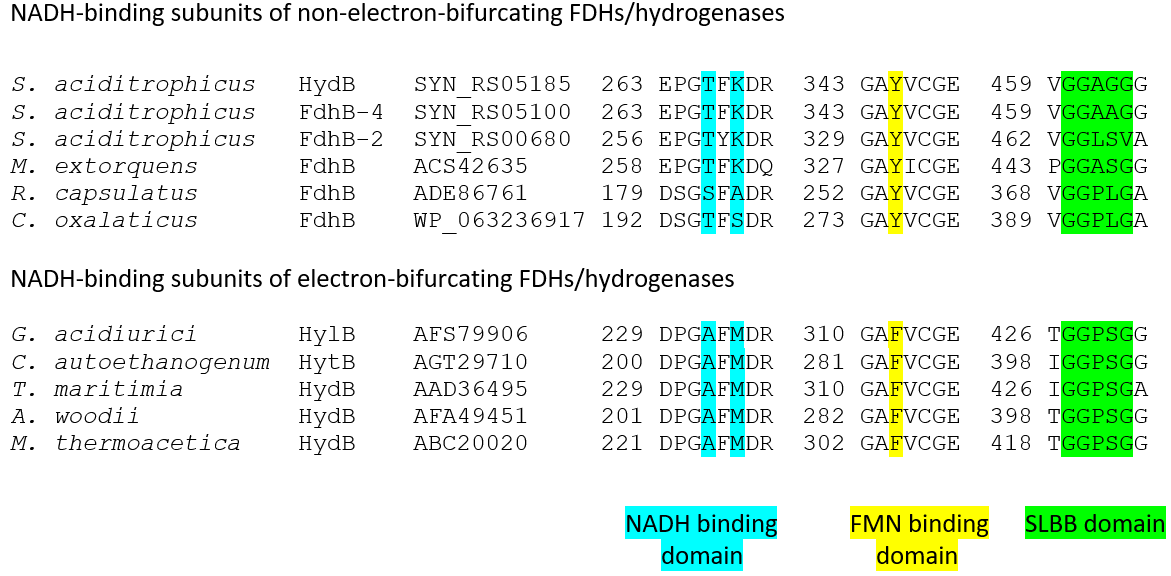


**Fig. S1** Distinguishing amino acid residues in the NADH/FMN-binding subunits of electron-bifurcating and non-electron-bifurcating FDHs/hydrogenases. The soluble NADH-dependent FDHs of *S. aciditrophicus* are highlighted by a blue frame. Other sequences were taken from Losey et al. 2020 (12).
