# Supplementary material for: Enoyl-Coenzyme A Respiration via Formate Cycling in Syntrophic Bacteria
Source: mBio. 2022 Feb 1;13(1):e03740-21. doi: 10.1128/mbio.03740-21 (PMC8805022; doi:10.1128/mbio.03740-21)
Supplement: TABLE S1 [file mbio.03740-21-st001.docx]

**Table S1** NuoF-like gene products in the genome of S. aciditrophicus. The HydB subunit of [Fe-Fe]-hydrogenase (SYN_RS05185) served as query sequence during BLAST application.

| **Gene product** | **Gene locus** | **Expect-value during BLAST search** | **Abundance in proteomes in crotonate grown cells** (31) | **Subcellular location** |
| --- | --- | --- | --- | --- |
| HydB subunit of  [Fe-Fe]-hydrogenase | SYN_RS05185 | 0.0 | +++ | cytoplasm |
| FdhB subunit of FDH-4 | SYN_RS05100 | 0.0 | +++ | cytoplasm |
| FdhB subunit of FDH-2 | SYN_RS00680 | 4e^–133^ | + | cytoplasm |
| BamH subunit of class II benzoyl-CoA reductase | SYN_RS00435 | 5e^–121^ | + | membrane/cytoplasm*^a^* |
| BamH subunit of class II benzoyl-CoA reductase | SYN_RS13430 | 5e^–117^ | - | membrane/cytoplasm*^a^* |
| BamH subunit of class II benzoyl-CoA reductase | SYN_RS14245 | 5e^–117^ | - | membrane/cytoplasm*^a^* |
| Subunit of Rnf complex | SYN_RS14175 | 1e^–07^ | +++ | membrane |

*^a^*Class II benzoyl-CoA reductases are membrane-associated, but are partially released to the soluble fraction during the preparation of cell extracts (32, 60)
