# Supplementary material for: Enoyl-Coenzyme A Respiration via Formate Cycling in Syntrophic Bacteria
Source: mBio. 2022 Feb 1;13(1):e03740-21. doi: 10.1128/mbio.03740-21 (PMC8805022; doi:10.1128/mbio.03740-21)
Supplement: FIG S2 [file mbio.03740-21-sf002.docx]

**Fig. S2** Absorption spectrum and reduction of Fd from *S. aciditrophicus*. **A,** Ultra-violet/visible spectrum of Fd from *S. aciditrophicus* as isolated from wild type cells with absorption maxima at 280 and 379 nm. **B,** Reduction of *S. aciditrophicus* Fd with 5 mM pyruvate, 0.5 mM CoA and soluble cell extract of *S. aciditrophicus* at 37 °C. Spectra were recorded in the direction of the arrow, at time points before 1, 2, 5 and 15 min after the addition of CoA.
