# Supplementary material for: Enoyl-Coenzyme A Respiration via Formate Cycling in Syntrophic Bacteria
Source: mBio. 2022 Feb 1;13(1):e03740-21. doi: 10.1128/mbio.03740-21 (PMC8805022; doi:10.1128/mbio.03740-21)
Supplement: FIG S3 [file mbio.03740-21-sf003.docx]

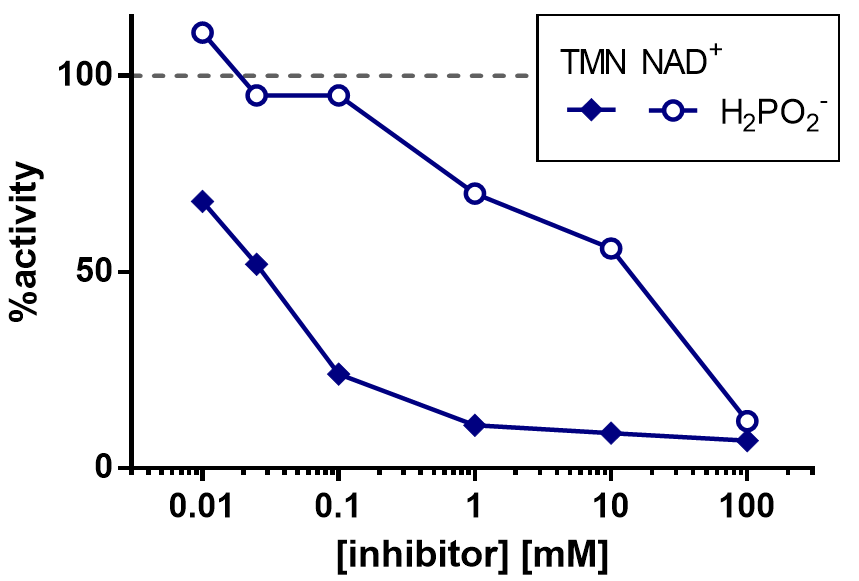


**Fig. S3** Inhibition of FDH activities by hypophosphite. Effect of increasing hypophosphite concentrations on *in vitro* TMN- (2,3,5-trimethyl-naphthoquinone, mFDH) or NAD^+^-dependent (sFDH) activities as measured in washed membranes or soluble cell extract, respectively. 100% activity refers to 1.14 U g^–1^ cell wet weight for TMN dependent mFDH activity and 1.55 U g^–1^ cell wet weight for NAD^+^ dependent sFDH activity.
